# Case report: Ethylene glycol intoxication presenting as a mimic of acute stroke: a report of three cases

**DOI:** 10.3389/fstro.2023.1233229

**Published:** 2023-08-02

**Authors:** Máté Héja, László Oláh

**Affiliations:** Department of Neurology, Faculty of Medicine, University of Debrecen, Debrecen, Hungary

**Keywords:** acute stroke, ethylene glycol, stroke mimic, intravenous thrombolysis, intoxication

## Abstract

Stroke is a major cause of death and disability presenting with acute focal neurological symptoms of vascular origin. Several other disorders may cause symptoms similar to a stroke, referred to as stroke mimics. The misdiagnosis of stroke mimics may lead to potentially harmful treatments, including thrombolysis. Intoxication is a rare, but possible, cause of stroke mimic. We present three cases of ethylene glycol poisoning presenting as an acute stroke mimic within the time window of thrombolytic therapy. Two of three patients (a 54-year-old male and a 78-year-old male) had dysarthria, nystagmus, and truncal ataxia on admission. The third patient with a history of chronic alcoholism presented after an epileptic seizure with mixed aphasia and confusion. Non-contrast cerebral computed tomography and computed tomography angiography were negative in all three cases. As stroke could not be excluded in any of the patients, thrombolysis was performed. However, after some hours, two of the three patients developed agitation, somnolence, and hyperventilation. One patient's consciousness deteriorated rapidly, and he became comatose and tetraplegic. A blood gas analysis showed acidosis in two of the three patients, and toxicological screening revealed ethylene glycol intoxication in all three cases. Due to the appropriate treatment, two of the three patients became symptom-free; however, one of the three patients died. Our cases show that ethylene glycol intoxication in its early phase may mimic acute stroke, resulting in unnecessary thrombolytic therapy. Symptoms not characteristic of a stroke, such as hyperventilation, agitation, and disturbance of consciousness, may appear later and warn of intoxication. The final diagnosis of ethylene glycol intoxication can be established by severe metabolic acidosis and toxicological screening. Close monitoring of symptoms might contribute to the early recognition of ethylene glycol intoxication and its effective treatment.

## 1. Introduction

Cerebrovascular diseases are the second-most common cause of death and the leading cause of disability worldwide (GBD 2016 Stroke Collaborators, 2019). Stroke is classically defined as the rapid development of clinical signs of focal (or global) disturbance of cerebral function, lasting more than 24 h or leading to death, with no apparent cause other than that of vascular origin (Aho et al., 1980). There are several other non-vascular diseases that may mimic stroke symptoms, including both neurological and non-neurological causes, known as stroke mimics (Long, 2016). The most common stroke mimics are seizures; peripheral vestibular lesions; migraine; brain tumors; metabolic disorders, for example, hypoglycemia; and functional disorders (Fernandes et al., 2013). Stroke mimics are often indistinguishable from an actual stroke, and misdiagnosis ranges from 5 to 31% of patients depending on the diagnostic methods (Long, 2016). Intravenous recombinant tissue plasminogen activator (rt-PA) is an effective and safe treatment for acute ischemic stroke (AIS) administered within 4.5 h after the onset of symptoms (Hacke et al., 2008; Wahlgren et al., 2008). In the acute setting, after excluding cerebral hemorrhage using brain computed tomography (CT), the diagnosis of AIS is mainly clinical. Moreover, due to the short time window in AIS, there is no time for detailed investigations. Therefore, it is not surprising that a subset of patients presenting with focal neurological deficits are misdiagnosed as stroke and treated with rt-PA. The most feared complication of intravenous thrombolysis (IVT) is intracranial hemorrhage, which is rare but not insignificant. Based on the literature, the rate of stroke mimics treated with rt-PA varies from 1 to 20% (Winkler et al., 2009; Lee et al., 2022), and the rate of symptomatic intracranial hemorrhage (sICH) ranges from ~0.5 to 1% in these patients (Merino et al., 2013; Zinkstok et al., 2013; Tsivgoulis et al., 2015).

Intoxication is a rare, but possible, cause of stroke mimic, accounting for ~2% of cases (Fernandes et al., 2013). Here, we detail three cases of ethylene glycol poisoning presenting as an acute stroke mimic within the time window of thrombolytic therapy. To the best of our knowledge, this is the first report of ethylene glycol intoxication that mimicked stroke symptoms and led to IVT.

## 2. Case description

### 2.1. Case 1

**Figure 1:**
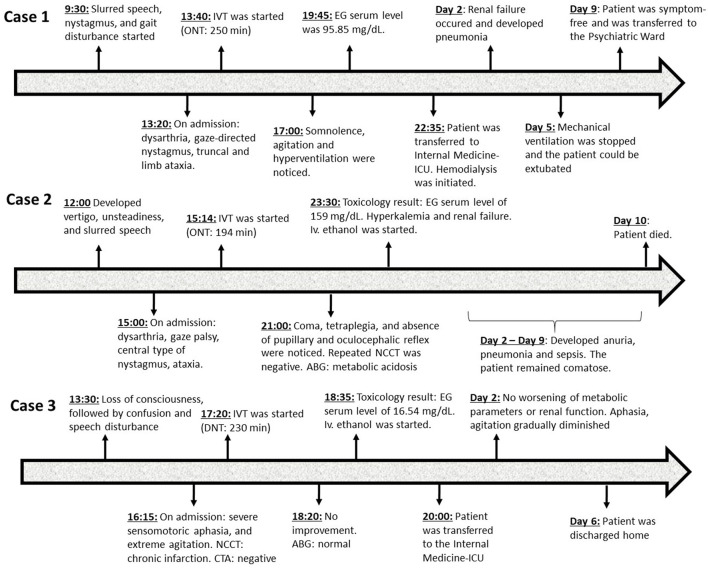
Timeline of the three cases. NCCT, non-contrast cerebral computed tomography; CTA, computed tomography angiography; IVT, intravenous thrombolysis; ONT, onset-to-needle time; ABG, arterial blood gas; EG, ethylene glycol; ICU, intensive care unit.

A 54-year-old man, who was a current smoker with no major illnesses in his medical history, was transferred to our department by the paramedics as a stroke alert, with a sudden onset of slurred speech, nystagmus, and gait disturbance that started in the morning (9:30 a.m.). On admission (1:20 p.m.), his neurological examination revealed severe dysarthria, horizontal and vertical gaze-directed nystagmus, and severe truncal and right-sided limb ataxia (National Institutes of Health Stroke Scale (NIHSS): 4 points). He was fully alert, afebrile, moderately hypertensive (165/90 mmHg), and slightly tachycardic (103/min, sinus rhythm). Pulse oximetry showed a hemoglobin saturation of 98% on room air. Non-contrast cerebral computed tomography (NCCT) imaging showed no signs of bleeding or acute ischemic lesion. CT angiography (CTA) imaging showed a hypoplastic vertebral artery on the left side, but no occlusion was observed on major intracranial arteries. His laboratory values showed no significant changes in routine laboratory parameters. Based on the anamnesis taken from the patient, the referral from the paramedics, and his clinical symptoms, a vertebrobasilar territory stroke was suspected, and IVT was started (1:40 p.m., door-to-needle time: 20 min, onset-to-needle-time: 250 min). A total dose of 73 mg of alteplase was administered. The patient's neurological status did not change during thrombolysis. However, late in the afternoon (5 p.m.), somnolence, agitation, and hyperventilation were noticed. Due to the disturbance of consciousness, cranial CT imaging was repeated to exclude intracranial hemorrhage, but it was negative again. Arterial blood gas (ABG) analysis disclosed severe metabolic acidosis (pH: 7.03, PaCO_2_ 21 mmHg, serum bicarbonate 5.9 mMol/L, base excess −22.7 mMol/L, anion gap: 24.7 mMol/L) (Table 1). The patient's agitation increased, requiring sedation, endotracheal intubation, and mechanical ventilation. Intravenous sodium bicarbonate was administered to correct the acidosis. Meanwhile, the patient's wife arrived and said that the patient had appeared depressed in the past few weeks and had mentioned suicidal thoughts several times, which increased the probability of an intoxication. Based on the metabolic acidosis, we suspected methanol or ethylene glycol poisoning, and an infusion of 10 g of 10% ethanol was started immediately. Within the next hours, a toxicological screening revealed an ethylene glycol serum concentration of 95.85 mg/dL, with toxic levels defined as >20 mg/dL and “possibly fatal” levels defined as >30 mg/dL (Table 2). Later, the patient was transferred to the intensive care unit of the internal medicine department, and urgent hemodialysis was initiated. Within 48 h, his renal function deteriorated [from a glomerular filtration rate (GFR) of 90 mL/min/1.73 m^2^ to 45 mL/min/1.73 m^2^ GFR] (Table 2), and the examination of urine sediment was positive for calcium oxalate crystals. The patient became febrile, and a chest X-ray showed pneumonia; therefore, intravenous antibiotics were started. His condition improved significantly in the following days. Control NCCT imaging 24 h after rt-PA treatment showed no abnormalities. The control toxicology test on the 2nd day no longer confirmed the presence of ethylene glycol in the blood, and the pH returned to normal; therefore, hemodialysis could be discontinued. Five days after his admission, mechanical ventilation was stopped, and the patient could be extubated. After he regained consciousness, he admitted to drinking antifreeze liquid with suicidal intent (~0.5–1 dL) a few hours before his admission. On day 9, he was transferred to the psychiatric ward for further treatment. At discharge, he was symptom-free. At discharge, he was symptom-free. Figure 1 shows the timeline of the three cases.

**Table 1:** Arterial blood gas (ABG) parameters of the three cases on admission.

**ABG parameters on admission**	**Case 1**	**Case 2**	**Case 3**
pH	7.03	< 6.8	7.45
PaO_2_ (mmHg)	74	83	87
PaCO_2_ (mmHg)	21	24	29
${\text{HCO}}_{3}^{-}$ (mmol/L)	5.9	< 3	20.2
${\text{HCO}}_{3}^{-}$std (mmol/L)	7.3	–	20.8
BE (mmol/L)	−22.7	−27.6	−2.5
AG (mmol/L)	24.7	28	15.6
Lactic acid (mmol/L)	>15	>15	1.4
Ca^2+^ (mmol/L)	1.27	0.84	0.24

BE, base excess; AG, anion gap.

**Table 2:** Laboratory values on admission and at 48 h.

**Laboratory values**	**Case 1**	**Case 2**	**Case 3**
**On admission**			
Sodium (mmol/L)	139	143	**122**
Potassium (mmol/L)	4.9	4.8	**5.5**
Glucose (mmol/L)	5.8	5.6	4.5
Creatinine (mg/dL)	0.72	0.95	0.53
GFR (mL/min)	>90	76	>90
LDH (U/L)	**224**	217	182
AST (U/L)	32	17	21
ALT (U/L)	40	16	9
GGT (U/L)	**116**	14	10
CRP (mg/L)	1.12	2.3	9.2
WBC (G/L)	10.75	8.43	**12.47**
Hemoglobin (g/L)	170	150	148
Platelet (G/L)	306	197	271
INR	0.98	1.0	0.96
EG (mg/dL)	**95.85**	**159**	**16.54**
**At 48 h**			
Sodium (mmol/L)	149	**152**	135
Potassium (mmol/L)	**5.4**	**5.9**	3.9
Glucose (mmol/L)	**6.4**	**6.5**	**7.0**
Creatinine (mg/dL)	**1.69**	**2.56**	0.57
GFR (mL/min)	**45**	**23**	>90
LDH (U/L)	**273**	**422**	**225**
AST (U/L)	23	37	**53**
ALT (U/L)	27	20	16
GGT (U/L)	**93**	**21**	11
CRP (mg/L)	**51.9**	**124**	**42.3**
WBC (G/L)	**14.54**	**19.87**	7.49
Hemoglobin (g/L)	141	152	129
Platelet (G/L)	191	**141**	208
INR	1.05	1.15	1.15
EG (mg/dL)	0	0	0

Abnormal values are bolded.

GFR, glomerular filtration rate; LDH, lactate dehydrogenase; AST, aspartate aminotransferase; ALT, alanine aminotransferase; GGT, gamma-glutamyl transferase; CRP, C-reactive protein; WBC, white blood count; INR, international normalized ratio; EG, ethylene glycol.

### 2.2. Case 2

A 78-year-old man with a history of hypertension, diabetes mellitus, and Parkinson's disease was admitted as a thrombolysis candidate with an ictal onset of vertigo, unsteadiness, and speech disturbance. According to his wife, he was asymptomatic in the morning on the day of admission, then at noon (12:00), he was noted to have difficulties in standing and walking without assistance, and his speech was slurred. The patient denied alcohol consumption or taking sedatives. On arrival (3 p.m.), horizontal gaze-directed nystagmus, right-sided horizontal gaze palsy, severe dysarthria, mild ataxia in all four extremities, and severe truncal ataxia causing an inability to walk were found (NIHSS: 5 points). At baseline, he was vigilant and fully oriented, and his vital parameters were in the normal range. NCCT and CTA imaging was negative. Laboratory screening on admission (including renal function) did not reveal significant abnormalities (Table 2). Since there were no exclusion criteria, thrombolysis was performed (onset-to-needle time: 194 min). After thrombolysis, his neurological symptoms did not improve. Moreover, a few hours later (9 p.m.), the patient's condition deteriorated rapidly and he lost consciousness, requiring intubation and mechanical ventilation. At that time, he was comatose and tetraplegic and showed bilateral Babinski signs, and his oculocephalic and pupillary light reflexes were absent, resembling an acute basilar artery occlusion or brainstem hemorrhage. NCCT and CTA imaging was repeated, but no signs of bleeding or large vessel occlusion were found. ABG analysis revealed extremely severe metabolic acidosis (pH < 6.8), an unmeasurable high lactate level (>15 mMol/L), low serum bicarbonate (< 3 mMol/L) level, and a wide anion gap (28 mMol/L) (Table 1). Despite several fluid boluses and sodium bicarbonate administration, the acidosis and lactate levels did not improve, and the patient became hemodynamically unstable, requiring continuous catecholamine infusion to maintain blood pressure. An urgent toxicological screening proved ethylene glycol intoxication with a serum ethylene glycol level of 159 mg/dL. Repeated laboratory tests (9 h after his admission) showed a sodium level of 147 mMol/L, a potassium level of 5.9 mMol/L, a creatinine level of 1.69 mg/dL, and GFR of 38 ml/min/1.73 m^2^ (Table 2). The patient was transferred to the intensive care unit of the internal medicine department, and ethanol infusion was started immediately accompanied by continuous renal replacement therapy (CRRT). A continuous infusion of bicarbonate was maintained to alkalinize the urine, and thiamine and pyridoxine were also given intravenously to facilitate the conversion of glycolate into less toxic metabolites. Ethylene glycol levels and blood pH were monitored regularly. When the pH returned to normal and ethylene glycol levels remained < 20 mg/dL, ethanol was stopped. Despite the aforementioned therapy, the patient became completely anuric, and his renal function continued to deteriorate, with elevations in creatinine over the next 10 days to a peak of 6.2 mg/dL. Urine analysis revealed calcium oxalate crystals. On the 5th day after admission, pneumonia developed with markedly elevated inflammatory markers, which required the administration of broad-spectrum antibiotics. Despite the withdrawal of sedation, the patient remained comatose. Unfortunately, he died on the 10th day after admission. We were informed later that the patient's mood had been depressed lately and that, although he had not mentioned suicidal thoughts, an empty bottle of antifreeze liquid was found in his garage.

### 2.3. Case 3

A 54-year-old man was transported to our department with a suspicion of an epileptic seizure. His medical history was significant for hypertension, chronic alcoholism, alcohol intoxication, delirium, and prior right-hemispheric ischemic stroke. According to his mother, the patient lost consciousness for a few minutes at 1:30 p.m. on the day of admission. After that, he was confused and disoriented, and his speech was unintelligible. Recent alcohol consumption was not reported. In addition to the moderate-severe left-sided spastic hemiparesis and Babinksi sign due to the previous stroke, severe mixed aphasia and extreme agitation were found during the physical examination (NIHSS: 7 points). To treat his agitation, clonazepam and tiapride were administered intravenously. No direct or indirect signs of convulsion were detected. NCCT imaging showed chronic infarction in the right internal capsule, but neither hemorrhage nor hyperacute ischemic lesion was described. CTA imaging showed no evidence of arterial occlusion or stenosis in the intracranial vessels. On admission, laboratory parameters were significant for mild hyponatremia (122 mMol/L) (Table 2). Since there were no exclusion criteria for intravenous thrombolysis, we decided to administer rt-PA in the standard dose of 0.9 mg/kg (5:20 p.m., onset-to-needle time: 230 min). His neurological status did not change during the thrombolysis. When considering potential alternative diagnoses, alcohol intoxication could not be excluded; therefore, blood was sent for toxicological examination. Although the toxicological screening was negative for ethanol, it revealed ethylene glycol intoxication, with an ethylene-glycol serum level of 16.54 mg/dL (6:35 p.m.). An ABG analysis was obtained, but it showed physiological acid–base parameters (Table 1). The patient was admitted to the intensive care unit of the internal medicine department, where intravenous ethanol was administered. His renal function did not show any worsening, so hemodialysis was not indicated. The next day, there was no detectable amount of ethylene glycol in his blood. The patient's aphasia and agitation gradually diminished. Hyponatremia was considered to be a consequence of chronic alcoholism and was corrected slowly with saline infusions. On the third day of hospitalization, he was able to walk with minor assistance and was able to take care of himself. He denied consuming antifreeze intentionally. On the 6th day after admission, he was discharged home with residual neurological symptoms consistent with his previous condition.

## 3. Discussion

Stroke is characterized by suddenly developed focal neurological symptoms of vascular origin. Diagnosing a stroke can be challenging because there are multiple diseases that may mimic its presentation. When acute stroke patients arrive directly at the neurology department, as in our institution, neurologists have to face this differential diagnostic challenge. In this report, we have described patients presenting with a rare cause of stroke mimic caused by ethylene glycol consumption.

Ethylene glycol is a colorless, odorless, sweet-tasting toxic alcohol most commonly found in antifreeze liquid for car engines and hydraulic brake fluids. Poisoning can be accidental or intentional, motivated by a suicide attempt. Although ethylene glycol intoxication is uncommon in medical practice, it is crucial to recognize because early diagnosis and prompt treatment can prevent severe morbidity and mortality (Hess et al., 2004). Clinical manifestation of acute toxicity includes central nervous system depression, cardiopulmonary symptoms, and renal insufficiency (Hess et al., 2004).

In our catchment area, all potential stroke patients are referred by paramedics to the neurologist on duty. The neurologist obtains information about the patient during the referral. Moreover, the patient's data can be retrieved from the national electronic health system that contains the patient's health data (e.g., major previous diseases, medications taken regularly, etc.). All potential stroke patients arrive directly at the CT laboratory where the initial examination and imaging are performed. According to the current guidelines, NCCT and CTA imaging is performed and evaluated immediately by a radiologist on duty. Depending on the signs and symptoms and the CT findings, the patients are transferred to the neurological intensive care unit after the CT examination, where trained nurses prepare the patients for thrombolytic therapy. Body weight, blood pressure, bedside blood glucose, and international normalized ratio measurements are performed using point-of-care devices; meanwhile, blood sampling is sent for urgent laboratory examination. BGA analysis does not belong to the routine procedures before IVT. If the history, physical examination, and imaging suggest ischemic stroke and the patient is otherwise eligible for rt-PA treatment, IVT is started. Generally, physicians do not wait for detailed laboratory results before thrombolytic therapy unless obvious warning signs are observed. Neurological status, signs of potential side effects (allergic reactions, minor or major bleeding), pulse, blood pressure, temperature, and oxygen saturation are monitored continuously according to the guidelines.

Our cases demonstrated that ethylene glycol poisoning can mimic the symptoms of an acute stroke. Acute alcohol intoxication is a frequently described vertebrobasilar stroke mimic as the two diseases share common symptoms of dysarthria, nystagmus, and gait disturbance (Arokszallasi et al., 2019; Hassing et al., 2019). The most common initial symptoms of ethylene glycol poisoning are the same as those of alcohol intoxication and vertebrobasilar stroke, including slurred speech, ataxia, and disturbance of consciousness (Davis et al., 1997). In line with the literature data, two of our three patients presented with the central type of nystagmus, severe dysarthria, and ataxia. With these patients, there was no further information to suggest other etiologies. Before the initiation of the rt-PA treatment, the patients' relatives were called, but they did not provide any information indicating intoxication. In our third patient, the differentiation of stroke and epilepsy posed a diagnostic challenge. The previous stroke and the chronic alcoholism in his past medical history, as well as the uncertain loss of consciousness at presentation and the subsequent aphasia and agitation, raised the suspicion of an epileptic seizure and postictal state, which may occur in the early phase of ethylene glycol intoxication (Hess et al., 2004). Although epilepsy is one of the most common stroke mimics, an epileptic seizure can rarely be the initial manifestation of stroke (Rolak et al., 1992). In addition, confusion and agitation may present if the stroke affects the non-dominant inferior parietal lobe, the non-dominant temporal gyrus, or the occipital lobe (Anathhanam and Hassan, 2017). According to the European Stroke Organization's latest guideline, for patients with AIS of < 4.5 h and who have seizures at the time of stroke onset, IVT is suggested (Berge et al., 2021). Since the patient was within the thrombolytic time window, had severe symptoms, and had no exclusion criteria for IVT, we decided to administer rt-PA. However, due to the atypical symptoms, an alternative diagnosis of intoxication was also considered; therefore, a blood sample was sent to toxicology for testing during the thrombolysis. ABGs in this patient did not indicate metabolic derangement, further complicating the establishment of the correct diagnosis. Due to the low rate of complications in stroke mimics treated with thrombolysis, we decided on IVT and performed further diagnostics tests during the treatment.

Differentiation between stroke and epilepsy with postictal hemiparesis and/or aphasia might be rather difficult in the hyperacute stage of the disease. Cerebral CT imaging usually does not help, and CTA imaging might also be negative in both diseases. Diffusion-weighted imaging, the most sensitive magnetic resonance imaging (MRI) method for cerebral ischemia (Chalela et al., 2007), could be useful, but MRI is not everywhere and not always available, and motion artifacts may also limit its evaluation. Moreover, in an acute situation when “time is brain” and rt-PA treatment requires specific time criteria, sometimes the treatment cannot be delayed by time-consuming examinations. Therefore, the general rule is that, if the clinical examination strongly suggests stroke, the worst should be assumed, and thrombolysis should be performed. In addition to MRI, perfusion computed tomography can also be a useful diagnostic tool in patients with stroke-like symptoms by providing information about cerebral blood flow (CBF), cerebral blood volume (CBV), and mean transit time (MTT). Since the ictal focus due to its high metabolic demand is hyperperfused, an increased CBF and CBV and a reduced MTT support a seizure rather than a stroke (Van Cauwenberge et al., 2018). However, in the postictal state, a hypoperfusion pattern may also appear, which would not help differentiate ischemic stroke and epilepsy. At the time when our patients were treated, CT perfusion imaging was not available in our department (Hedna et al., 2012).

To the best of our knowledge, this is the first report in the literature of ethylene glycol poisoning that not only mimicked acute stroke but also led to thrombolytic treatment. All of our patients were referred by paramedics as a stroke alert within the time window of thrombolysis, and no evidence of intoxication was mentioned on admission for any of the patients. If the history and symptoms suggest ischemic stroke within the time window and imaging excludes cerebral hemorrhage and does not indicate another intracranial disease, thrombolysis should be considered if the patient is otherwise eligible for rt-PA treatment. There were no exclusion criteria in any of our cases, and since our presumptive diagnosis was acute stroke and the patients had disabling symptoms, thrombolysis was performed. In our first and second patients, the symptoms on admission were typical for vertebrobasilar stroke without any other alternative diagnosis. In their case, the possibility of a stroke mimic arose only after the thrombolysis was completed. As we discussed earlier, the third patient's case was more complicated. The preceding loss of consciousness that raised the suspicion of an epileptic seizure was considered a stroke mimic, and extreme agitation is not typical in ischemic stroke. However, as thrombolysis is an effective therapy for ischemic stroke (National Institute of Neurological Disorders Stroke rt-PA Stroke Study Group, 1995) and a stroke mimic is associated with no or minimal risk of intracranial hemorrhage (Artto et al., 2012), it is not recommended to exclude patients who are potential candidates for rt-PA treatment based on the sole concern that their neurological symptoms may be attributed to a stroke mimic (Tsivgoulis et al., 2011). Our cases underline this statement because none of our patients suffered intracranial hemorrhage after thrombolysis, nor did they have any other disadvantages from the administration of rt-PA. Since IVT is not a harmless treatment, we attempt to avoid thrombolytic therapy for stroke mimics. However, in centers where the MRI machine is in another building, and NCCT and CTA imaging tests are routine before the administration of IVT, additional multimodal imaging may cause a significant delay in treatment. The results from the International Stroke Trial 3 confirmed that stroke patients benefit most from an early start of IVT (Sandercock et al., 2008). Based on the literature, the rate of sICH after IVT is very low in stroke mimics, and stroke mimics were more likely to experience an excellent outcome at 3 months compared with patients with AIS (Zinkstok et al., 2013). In our opinion, although efforts should be made to avoid IVT in stroke mimics, rapid treatment is likely more beneficial than adding extensive exams to rule out mimics in daily clinical practice.

Our observations suggest that symptoms of the first phase of ethylene glycol intoxication may overlap with the symptoms of an acute stroke, which may delay the diagnosis of this life-threatening condition. However, close monitoring during and after thrombolysis can facilitate the early recognition of the disease underlying the stroke mimic. As our cases have shown, the first symptoms of ethylene glycol poisoning, including nystagmus, dysarthria, and truncal ataxia, are also common in ischemic stroke. However, later symptoms, such as hyperventilation, agitation, and disturbance of consciousness are no longer characteristic of stroke and may draw attention to a stroke mimic. Besides the variable clinical symptoms, the hallmarks of ethylene glycol intoxication are (1) the presence of metabolic acidosis; (2) an elevated anion or osmolar gap; (3) positive findings for calcium oxalate crystals in the urine; and (4) toxicological detection of ethylene glycol in the serum or urine (Iqbal et al., 2022). It is important to recognize those alarming signs that may warn of intoxication during the observation of patients. Despite the thrombolysis, none of our patients' neurological symptoms improved; however, progressive disturbance of consciousness, agitation, or hyperventilation developed during the course of the disease. ABG analyses revealed severe metabolic acidosis with a wide anion gap in Case 1 and Case 2. These findings raised the suspicion of intoxication with alcohol derivatives, which was also confirmed by the toxicological screening. Later, both patients had evidence of nephrotoxicity, which is also a characteristic feature of ethylene glycol intoxication, demonstrated by elevated creatinine levels and decreased GFR values. However, in Case 3, ABG was normal, and renal function was not impaired. Its explanation is probably that the serum ethylene glycol did not reach the toxic value and the prompt treatment prevented the formation of toxic metabolites. Quite to the contrary, in our second patient, whose serum level was a multiple of the toxic value, very severe metabolic changes and central nervous system depression developed.

A suspected stroke diagnosis in stroke mimics, even if wrong, may lead to rapid hospitalization and close monitoring. Because stroke requires emergency care, patients with stroke symptoms, caused by either stroke or stroke mimics, usually reach the hospital in a very short time. Moreover, if reperfusion therapy is performed, the patient is carefully monitored not only during the rt-PA treatment but also in the subsequent 24 h. It means that patients with stroke or stroke mimics are investigated and observed in the initial stage of the disease, allowing recognition of the first signs and symptoms, and thus early diagnosis and treatment. In our cases, this management led to early diagnosis of ethylene glycol intoxication and early start of the appropriate treatment resulting in a good clinical outcome in two of our three patients.

The importance of anamnesis should also be highlighted. A report of the ingestion of toxic agents makes the diagnosis obvious; however, the physician should ask specifically about drinking antifreeze or similar substances in cases of suspected toxicity. Often, the patient is unwilling or unable to give an accurate history. Ideally, a bottle of antifreeze or other solvent is found at the scene, but if not, the family, as well as the prehospital team, should be thoroughly questioned and instructed to search for potentially toxic substances. Previous suicidal ideations and a history of depression are also key information. At the presentation of our patients, this information was lacking, and it became known only after the diagnosis was made.

## 4. Conclusion

In conclusion, our observations showed that ethylene glycol intoxication in its early phase may mimic acute stroke and thus even lead to thrombolytic treatment, which has not been reported earlier. If focal neurological deficits are accompanied by agitation, hyperventilation, or disturbance of consciousness, an alternative diagnosis, including intoxication, should be considered. Although the treatment with IVT should not be delayed, in patients with atypical symptoms, a detailed and cautious evaluation should be performed. The differential diagnosis in these cases can be facilitated by ABG, toxicological screening, and modern multimodal imaging techniques.

## Data availability statement

The original contributions presented in the study are included in the article/supplementary material, further inquiries can be directed to the corresponding author.

## Ethics statement

Written informed consent was obtained from the individual(s) for the publication of any potentially identifiable images or data included in this article.

## Author contributions

MH was involved in clinical workup, manuscript preparation, editing, and submission. LO revised the manuscript critically for important content. All authors contributed to the article and approved the final manuscript.
